# Long-term effect of statins on the risk of new-onset osteoporosis: A nationwide population-based cohort study

**DOI:** 10.1371/journal.pone.0196713

**Published:** 2018-05-03

**Authors:** Tsung-Kun Lin, Pesus Chou, Ching-Heng Lin, Yi-Jen Hung, Gwo-Ping Jong

**Affiliations:** 1 Taoyuan Armed Forces General Hospital, Taoyuan, Taiwan, ROC; 2 Institute of Public Health and Community Medicine Research Center, National Yang-Ming University, Taipei, Taiwan, ROC; 3 Department of Medical Research, Taichung Veterans General Hospital, Taichung, Taiwan, ROC; 4 Department of Internal Medicine, Division of Endocrinology and Metabolism, Tri-Service General Hospital, School of Medicine, National Defense Medical Center, Taipei, Taiwan, ROC; 5 Division of Internal Cardiology, Chung Shan Medical University Hospital and Chung Shan Medical University, Taichung, Taiwan, ROC; Harvard Medical School, UNITED STATES

## Abstract

**Background:**

Several observational cohort and meta-analytical studies in humans have shown that statin users have a lower risk of fractures or greater bone mineral densities (BMD) than nonusers. However, some studies including randomized clinical trials have the opposite results, particularly in Asian populations.

**Objective:**

This study investigates the impacts of statins on new-onset osteoporosis in Taiwan.

**Methods:**

In a nationwide retrospective population-based cohort study, 45,342 subjects aged between 50–90 years having received statin therapy (statin-users) since January 1 2001, and observed through December 31 2013 were selected from the National Health Insurance Research Database of Taiwan. Likewise, 115,594 patients had no statin therapy (statin-non-users) were included as controls in this study. Multivariable Cox proportional hazards analysis for drug exposures was employed to evaluate the association between statin treatment and new-onset of osteoporosis risk. We also used the long-rank test to evaluate the difference of probability of osteoporosis-free survival.

**Results:**

During the 13-year follow-up period, 16,146 of all enrolled subjects (10.03%) developed osteoporosis, including 3097 statin-users (6.83%) and 13,049 statin-non-users (11.29%). Overall, statin therapy reduced the risk of new-onset osteoporosis by 48% (adjusted hazard ratio [HR] 0.52; 95% CI 0.50 to 0.54). A dose-response relationship between statin treatment and the risk of new-onset osteoporosis was observed. The adjusted hazard ratios for new-onset osteoporosis were 0.84 (95% CI, 0.78 to 0.90), 0.56 (95% CI, 0.52 to 0.60) and 0.23 (95% CI, 0.21 to 0.25) when cumulative defined daily doses (cDDDs) ranged from 28 to 90, 91 to 365, and more than 365, respectively, relative to nonusers. Otherwise, high-potency statins (rosuvastatin and atorvastatin) and moderate-potency statin (simvastatin) seemed to have a potential protective effect for osteoporosis.

**Conclusions:**

In this population-based cohort study, we found that statin use is associated with a decreased risk of osteoporosis in both genders. The osteoprotective effect of statins seemed to be more prominent with a dependency on the cumulative dosage and statin intensity.

## Introduction

Statins, known as hydroxymethylglutaryl-CoA (HMG-CoA) reductase inhibitors, have been widely used as cholesterol-lowering drugs and there is strong evidence for beneficial effects for patients at risks for cardiovascular diseases [1–3]. Their efficacy and safety have been well documented in many primary and secondary clinical trials. However, cumulative experience and evidence also revealed new adverse effects from statins such as new-onset diabetes, cognitive impairment, and dementia [4–6].

In addition to their well-known cholesterol-lowering properties and potential adverse effects, other advantageous pleiotropic effects of statins have been noticed. An interesting impact is their effect on bone metabolism. The possible connection between statins and bone health was first reported in 1999, when the authors discovered that statin increased bone formation through stimulating the production of bone morphogenic protein-2 (BMP-2) in rodent bone cells [7]. Recent studies have also demonstrated that statins inherit potential properties of both antiresorptive and anabolic effects including proliferation, differentiation, protection of osteoblasts, and reducing osteoclast formation [8–11].

Although several observational cohort or case-control studies in humans found that statin users had a lower risk of fractures or greater bone mineral densities (BMD) than nonusers [12–16], some studies reported conflicting results [17–19], particularly in Asian populations. For example, a Japanese study of patients with type-2 diabetes seemed to indicate a negative correlation between statin use and BMD [20]. Thus, post-hoc analyses of large-scaled randomized studies including LIPID, JUPITER, and the Scandinavian Simvastatin Survival Study (4S) also demonstrated no association between statin use and a reduction of bone fracture risk [21–23]. The potential source of the discrepancy among these studies might be widely varying and related to ethnicity or gender as well as dosage, duration, and the specific statin used. Therefore, the controversy over the connection between statins and bone health prompted us to conduct a nationwide population-based retrospective, long-term follow-up study in Taiwan to investigate the impacts of stratification of different statins on new-onset osteoporosis.

## Materials and methods

### Data source

We constructed the study using collected data from the Longitudinal Health Insurance Database (LHID). All the registration and claim data of these 1,000,000 individuals collected by the National Health Insurance program constitute the LHID. The 1,000,000 beneficiaries were randomly selected from the Taiwan National Health Insurance program (Taiwan NHI), which was a nationwide and single-payer health insurance program. The claim data in LHID contained a registry of beneficiaries, inpatient and outpatient files (recorded physician diagnosis by the International Classification of Diseases, Ninth Revision, Clinical Modification [ICD-9-CM]), and medical service. LHID was a de-identification database and the Taiwan government updated the database every year. This study was approved by the ethical review board of the Taichung Veterans General Hospital (approval number: CE13152B-3).

### Study population

**Fig 1:**
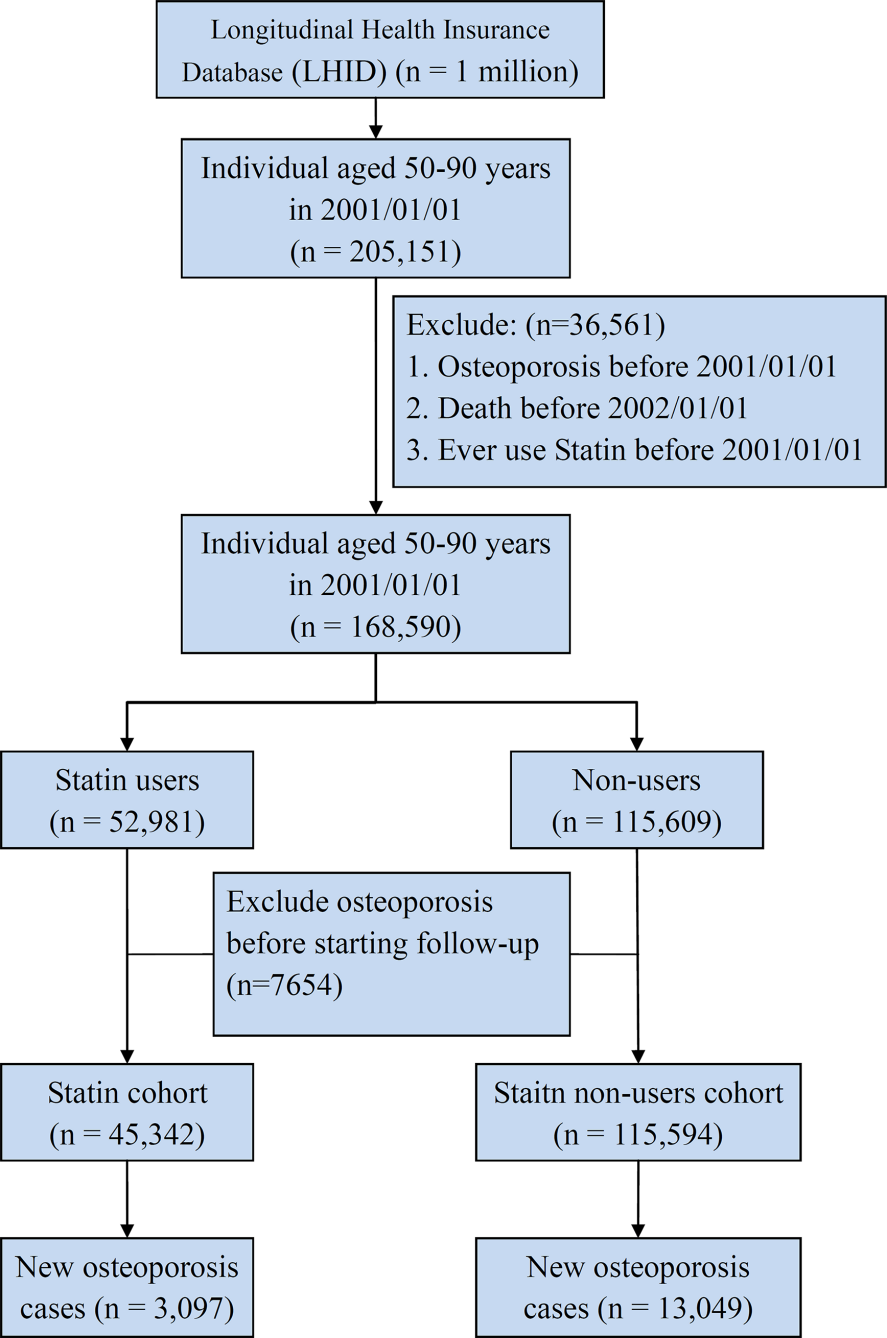
The study design flowchart of sample selection from National Health Insurance Research Database in Taiwan.

This study was designed as a retrospective population-based cohort study. Fig 1 shows a flow chart of the study population selection. We selected subjects aged 50–90 years as of January 1 2001 and then excluded subjects with a history of osteoporosis (ICD-9-CM 733.0) or with statin use before January 1 2001, or died before January 1 2002. The statin user cohort was formed by the subjects receiving statin treatment and the respective index date was set as the initial statin use day individually. On the other hand, the statin-non-user cohort was selected from subjects without statin use in the base population and randomly assigned a date after January 1, 2001 as an index date. The subjects who coincidentally had osteoporosis before the index date were excluded in both cohorts. Finally, we had a 45,342 statin-user cohort and a 115,594 statin-non-user cohort. The censor of the follow-up was considered when the subjects dismissed the health insurance, developed osteoporosis, or until December 31 2013.

Considering statins contain several subtypes such as simvastatin, lovastatin, pravastatin, fluvastatin, atorvastatin, rosuvastatin, and pitavastatin, the potency was taken account into the assessment for the effect upon osteoporosis risk [24]. To standardize statin exposure, we used the Anatomical Therapeutic Chemical (ATC) classification system to unify the statin exposure unit as the defined daily dose (DDD) and ATC code of statin was C10AA01-C10AA08.

We considered some comorbidities and medications as confounding factors in the current study. The baseline comorbidity was defined by subjects with a specific disease record before the index date. The comorbidity included alcohol related disorders (ALD, ICD-9-CM 291, 303, 305, 571.0, 571.1, 571.2, 571.3, 790.3, V11.3), chronic obstructive pulmonary disease (COPD, ICD-9-CM 490–492, 496), diabetes mellitus (DM, ICD-9-CM 250), hyperthyroidism (ICD-9-CM 242), liver cirrhosis (ICD-9-CM571.5, 571.6), and coronary artery disease (CAD, ICD-9-CM 410–414). We also included a history of hormone replacement therapy (HRT) for each subject before the index date in the confounding factors.

### Statistical analysis

The basic information of the study cohort was showed to include mean and standard deviation (SD) for age, presented as number and percentage for sex, along with baseline comorbidity and medication. To assess the difference between statin-users and statin-non-users, a t test was employed to test age difference, but the chi-square test was applied to assess the difference of sex, baseline comorbidity and medication. The probability of osteoporosis-free survival demonstrated that 1) statin users vs. non-users; and 2) stain-non-users vs. 4 potency-level of statin exposure and measured by Kaplan-Meier method. To test the curve difference, we used log-rank test. To evaluate the risk of osteoporosis between statin-users and statin-non-users, hazard ratios (HRs) and corresponding 95% confidence intervals (CIs) was estimated by single variable and multivariable Cox proportional hazard models. SAS 9.4 software (SAS Institute, Cary, NC, USA) was performed to compute the statistical analysis and R software (R Foundation for Statistical Computing, Vienna, Austria) was used to draw the survival curve. The significant level was set at less than 0.05 for two-side testing of *P* value.

## Results

### Baseline demographic status

In total, 45,342 statin-users and 115,594 statin-non-users with mean age 66.6±8.36 and 67.5±10.0 years, respectively, were enrolled for analysis. The ages of statin-users were younger and had more comorbidities such as diabetes, COPD, or CAD etc. when compared to statin-non-users (*P* < 0.0001). In females, statin-users have higher rate to receive hormone replacement therapy (HRT) concurrently (*P* < 0.0001) (Table 1).

**Table 1:** Baseline demographic status and comorbidity between statin users and non-users.

Variable	Overall			Female			Male		
	Non-users	Statin users	*P* value	Non-users	Statin users	*P* value	Non-users	Statin users	*P* value
	n = 115,594 (%)	n = 45,342 (%)		n = 47,784 (%)	n = 20,845 (%)		n = 67,810 (%)	n = 24,497 (%)	
Age, years (SD)^*^	67.5 (10.0)	66.6 (8.36)	<0.0001	66.9 (10.2)	66.3 (8.26)	<0.0001	67.9 (9.83)	66.8 (8.45)	<0.0001
**Gender**			<0.0001						
Female	47,784 (41.3)	20,845 (46.0)							
Male	67,810 (58.7)	24,497 (54.0)							
**Comorbidity**									
ALD	2,142 (1.85)	998 (2.20)	<0.0001	208 (0.44)	136 (0.65)	0.0002	1,934 (2.85)	862 (3.52)	<0.0001
COPD	39,376 (34.1)	16,595 (36.6)	<0.0001	14,271 (29.9)	7,024 (33.7)	<0.0001	25,105 (37.0)	9,571 (39.1)	<0.0001
DM	19,678 (17.0)	19,857 (43.8)	<0.0001	7,945 (16.6)	9,193 (44.1)	<0.0001	11,733 (17.3)	10,664 (43.5)	<0.0001
Hyperthyroidism	1,227 (1.06)	805 (1.78)	<0.0001	784 (1.64)	585 (2.81)	<0.0001	443 (0.65)	220 (0.90)	0.0001
Liver cirrhosis	3,279 (2.84)	590 (1.30)	<0.0001	1,069 (2.24)	210 (1.01)	<0.0001	2,210 (3.26)	380 (1.55)	<0.0001
CAD	26,633 (23.0)	17,864 (39.4)	<0.0001	10,745 (22.5)	7,683 (36.9)	<0.0001	15,888 (23.4)	10,181 (41.6)	<0.0001
**Medication**									
HRT	13,556 (11.7)	7,503 (16.6)	<0.0001	13,268 (27.8)	7,395 (35.5)	<0.0001			

*t test

ALD: alcohol related disorder; COPD: chronic obstructive pulmonary disease; DM: diabetes mellitus; CAD: coronary artery disease; HRT: hormone replacement therapy

### The effect of statins on new-onset osteoporosis

At the end of 13-year follow-up, 16,146 of all enrolled subjects (10.03%) developed osteoporosis, including 3,097 statin-users (6.83%) and 13,049 statin-non-users (11.29%). The statin-users tended to have a lower rate of developing osteoporosis at the end of follow-up than the statin-non-users (*P* < 0.0001).

Table 2 displays the results of Cox regression analysis of the baseline factors associated with the rate of new-onset osteoporosis. Cox proportional hazards regression (HR) analysis revealed that statin-users had significantly lower rate of new-onset osteoporosis when statin-non-users as a reference after adjusting for age, sex, and comorbidities (HR 0.52 (95% CI = 0.50–0.54, *P* < 0.0001)). In both males and females, statin-users also had significantly lower rates of new-onset osteoporosis than statin-non-users even further adjusting for HRT in female (HR 0.53 (95% CI = 0.49–0.58, in male, *P* < 0.0001); HR 0.52 (95% CI = 0.49–0.54, in female, *P* < 0.0001)).

**Table 2:** Adjusted hazard ratios of baseline factors for new-onset osteoporosis.

Variable	All		Female		Male	
	n = 160,936		n = 68,629		n = 92,307	
	HR (95% CI)^a^	*P* value	HR (95% CI)^b^	*P* value	HR (95% CI)^c^	*P* value
**Statin use**						
Non-users	ref		ref		ref	
Users	0.52(0.50–0.54)	<0.0001	0.52(0.49–0.54)	<0.0001	0.53(0.49–0.58)	<0.0001
Age, years (SD)^*^	1.03(1.03–1.03)	<0.0001	1.02(1.02–1.03)	<0.0001	1.04(1.04–1.05)	<0.0001
**Sex**						
Female	3.52(3.40–3.64)	<0.0001				
Male	ref					
**Comorbidity**						
ALD	1.03(0.88–1.19)	0.75	1.14(0.88–1.48)	0.32	1.02(0.85–1.23)	0.83
COPD	1.48(1.43–1.53)	<0.0001	1.36(1.31–1.42)	<0.0001	1.70(1.60–1.81)	<0.0001
DM	1.05(1.01–1.09)	0.02	1.04(0.99–1.09)	0.12	1.07(1.00–1.15)	0.04
Hyperthyroidism	1.06(0.94–1.20)	0.32	1.02(0.89–1.17)	0.77	1.20(0.88–1.64)	0.25
Liver cirrhosis	1.01(0.91–1.14)	0.81	0.97(0.83–1.12)	0.65	1.10(0.92–1.32)	0.30
CAD	1.20(1.16–1.25)	<0.0001	1.19(1.14–1.24)	<0.0001	1.20(1.13–1.28)	<0.0001
**Medication**						
HRT			1.17(1.12–1.22)	<0.0001		

^a^ Model adjusted for age, sex, ALD, COPD, DM, Hyperthyroidism, Liver cirrhosis and CAD

^b^ Model adjusted for age, ALD, COPD, DM, Hyperthyroidism, Liver cirrhosis, CAD and HRT

^c^ Model adjusted for age, ALD, COPD, DM, Hyperthyroidism, Liver cirrhosis and CAD

ALD: alcohol related disorder; COPD: chronic obstructive pulmonary disease; DM: diabetes mellitus; CAD: coronary artery disease; HRT: hormone replacement therapy

*t test

To clarify the effect between new-onset osteoporosis and statins, subgroup analysis was further performed. Table 3 shows that new-onset osteoporosis risks had a declining trend that paralleled when statin cDDDs increased (HR 1.03, 0.84, 0.56 and 0.23 in cDDDs <28 days, 28–90 days, 91–365 days and ≧366 days, respectively, *P* for trend < 0.0001). On the other hand, a significantly lower risk for new-onset osteoporosis was found in high-potency statins (rosuvastatin and atorvastatin) and moderate-potency statin (simvastatin), in comparison to statin-non-users [HR 0.43 (95% CI = 0.36–0.52, *P* < 0.0001); HR 0.68 (95% CI = 0.63–0.74, *P* < 0.0001) and HR 0.85 (95% CI = 0.76–0.94, *P* = 0.003, respectively)] (Table 4). Meanwhile, no significant osteoprotective effect was found in low-potency statins including lovastatin, pravastatin, and fluvastatin, etc. Table 5 showed the effect of different cDDD level in simvastatin, rosuvastatin and atorvastatin for new-onset osteoporosis risk. HRs for new-onset osteoporosis were 1.01 (95% CI = 0.83–1.22), 0.89 (95% CI = 0.75–1.06), 0.74 (95% CI = 0.59–0.93) and 0.46 (95% CI = 0.30–0.71) for < 28 cDDD, 28–90 cDDD, 91–365 cDDD and ≧366 cDDD in simvastatin, respectively. In atorvastatin, relative to statin non-users, the new-onset osteoporosis risk were 0.99 (95% CI = 0.86–1.14), 0.85 (95% CI = 0.75–0.97), 0.61 (95% CI = 0.52–0.70) and 0.28 (95% CI = 0.22–0.36) in < 28 cDDD, 28–90 cDDD, 91–365 cDDD and ≧366 cDDD, respectively. The results showed rosuvastatin users had a protect effect for new-onset osteoporosis in all level of cDDDs.

**Table 3:** Adjusted hazard ratios of statins cDDDs for new-onset osteoporosis.

cDDDs level	n	HR (95% CI)	*P* value
cDDDs			
Non-users	115594	ref	-
<28 DDDs	6420	1.03(0.95–1.11)	0.47
28–90 DDDs	8858	0.84(0.78–0.90)	<0.0001
91–365 DDDs	13501	0.56(0.52–0.60)	<0.0001
≥366 DDDs	16563	0.23(0.21–0.25)	<0.0001

Model adjusted for age, sex, ALD, COPD, DM, Hyperthyroidism, Liver cirrhosis, CAD and HRT

Abbreviations: cDDD, cumulative defined daily dose

**Table 4:** Adjusted hazard ratios of statins subtype for new-onset osteoporosis.

Statin status	n	HR (95% CI)	p-value
Subtype			
Simvastatin	3690	0.85(0.76–0.94)	0.003
Lovastatin	3244	1.08(0.99–1.18)	0.08
Pravastatin	1443	0.89(0.76–1.05)	0.18
Fluvastatin	1595	0.92(0.79–1.07)	0.28
Atorvastatin	9639	0.68(0.63–0.74)	<0.0001
Rosuvastatin	2985	0.43(0.36–0.52)	<0.0001

Model adjusted for age, sex, ALD, COPD, DM, Hyperthyroidism, Liver cirrhosis, CAD and HRT

**Table 5:** Adjusted hazard ratios of statins cDDDs for new-onset osteoporosis in simvastatin, atorvastatin and rosuvastatin users.

cDDDs level	Simvastatin			Atorvastatin			Rosuvastatin		
	n	HR (95% CI)	p-value	n	HR (95% CI)	p-value	n	HR (95% CI)	p-value
Non-users	115594	ref		115594	ref		115594	ref	
<28 DDDs	1103	1.01(0.83–1.22)	0.94	2190	0.99(0.86–1.14)	0.90	458	0.61(0.40–0.94)	0.02
28–90 DDDs	1318	0.89(0.75–1.06)	0.21	2884	0.85(0.75–0.97)	0.02	772	0.43(0.29–0.63)	<0.0001
91–365 DDDs	934	0.74(0.59–0.93)	0.008	2869	0.61(0.52–0.70)	<0.0001	932	0.56(0.41–0.76)	0.0001
≥366 DDDs	335	0.46(0.30–0.71)	0.0004	1696	0.28(0.22–0.36)	<0.0001	823	0.23(0.15–0.35)	<0.0001

Model adjusted for age, sex, ALD, COPD, DM, Hyperthyroidism, Liver cirrhosis, CAD and HRT

Abbreviations: cDDD, cumulative defined daily dose

Table 6 demonstrated the risk of new-onset osteoporosis between statin users and non-users cohort by follow-up duration. The results reveled that HR for new-onset osteoporosis was 0.49 (95% CI = 0.47–0.52) in statin users cohort relative to non-users cohort in Year 0–7 and HR was 0.83 (95% CI = 0.73–0.95) after Year 7. In female, the statin users cohort was significantly lower risk of new-onset osteoporosis than the non-users cohort only in Year 0–7 (HR = 0.49, 95% CI = 0.46–0.51). In male, the statin users cohort had a lower risk of new-onset osteoporosis relative to the non-users cohort both in duration Year 0–7 (HR = 0.51, 95% CI = 0.47–0.56) and after Year 7 (HR = 0.74, 95% CI = 0.59–0.93).

**Table 6:** Hazard ratios for osteoporosis in statin users cohort relative to non-users cohort by follow-up duration.

Follow-up duration	Statin use	All			Female			Male		
		n	HR (95% CI)^a^	*P* value	n	HR (95% CI)^b^	*P* value	n	HR (95% CI)^c^	*P* value
Year 0–7	Non-users	115594	ref		47784	ref		67810	ref	
	Users	45342	0.49(0.47–0.52)	<0.0001	20845	0.49(0.46–0.51)	<0.0001	24497	0.51(0.47–0.56)	<0.0001
After Year 7	Non-users	39888	ref		16961	ref		22927	ref	
	Users	17206	0.83(0.73–0.95)	0.005	8162	0.88(0.76–1.03)	0.11	9044	0.74(0.59–0.93)	0.009

^a^ Model adjusted for age, sex, ALD, COPD, DM, Hyperthyroidism, Liver cirrhosis and CAD

^b^ Model adjusted for age, ALD, COPD, DM, Hyperthyroidism, Liver cirrhosis, CAD and HRT

^c^ Model adjusted for age, ALD, COPD, DM, Hyperthyroidism, Liver cirrhosis and CAD

ALD: alcohol related disorder; COPD: chronic obstructive pulmonary disease; DM: diabetes mellitus; CAD: coronary artery disease; HRT: hormone replacement therapy

## Discussion

Our main findings of this retrospective and large-scaled cohort study with 13 years of follow-up indicated that therapeutic doses of statins seem to have an osteoprotective effect that prevents patients from occurrences of osteoporosis with a 47% reduction in males and 48% in females. High-potency statins (atorvastatin and rosuvastatin) and moderate-potency statins (simvastatin) are more effective in decreasing the new development of osteoporosis. No significant association between new-onset osteoporosis and low-potency statins (lovastatin, pravastatin, and fluvastatin) was observed. Furthermore, the protective effect of osteoporosis was enhanced in parallel with the cumulative doses of statins.

The effects of statins on osteoporosis, BMD, the fracture risk, and the biomarkers of bone turnover have been reported in the literature with different study designs. Most studies, including the studies from European population, conducted with the design as observational, case-control, prospective cohort, and a meta-analysis format displayed the beneficial effects of bone metabolism in statin-users [13, 14, 16, 25–27]. One recent meta-analytical study including two large-scaled RCTs (LIPID and JUPITER) indicated that statin treatment was associated with a decreased risk of overall hip fractures and increased BMD at hips and lumbar spines [27]. In our current study, we also found that statins have a potentially beneficial effect to reduce the incidences of osteoporosis even with adjustment for comorbidities and HRT in female, although the BMD and bone fractures were not assessed. However, some studies and post-hoc analyses of RCTs did not reveal the positive effect of bone fracture risks. In JUPITER, LIPID, 4S, and HPS trials in patients with high cardiovascular risks or diabetes using rosuvastatin, pravastatin, and simvastatin, respectively, statins were not associated with a decreased risk for fracture [21–23]. Among postmenopausal women enrolled from a prospective study of Women’s Health Initiative observational study, statin therapy was also reported to neither improve bone density nor reduce fracture risk [17]. Several reasons were proposed to explain the inconsistency among effects of statin on bone metabolism. Different ethnicity of patients, exposure duration of distinct statins, concurrent medications, and inadequate adjustment for confounders, among others, were probably contributive to this discordance.

In addition, the greater reduction of osteoporosis risk derived from statin therapy was observed in our study. In both males and females, statin-users had similarly significant decreases of new-onset osteoporosis in comparison with statin-non-users, by further adjusting for HRT in females (HR 0.53 in males and HR 0.52 in females). The osteoporosis risk had a trend to be declined in parallel with increased cumulative doses of statin (HR 1.03 to 0.23 in cDDDs <28 days to ≧366 days). In a recent meta-analytical study, the odds ratio (OR) for risk reduction of bone fracture on statin therapy ranged from 0.48 to 1.10 and overall OR 0.81 (95% CI 0.73–0.89) with at least a one-year treatment period [27]. Regardless of different target outcomes on statin therapy, the association in our study is stronger than those published previously. We speculated that long-termed exposure to statins might only provide a small contribution. This was probably and mainly due to an inadequate adjustment of health-seeking behavior, calcium intake, and other residual unexplored confounding factors.

From the available data of the majority of experimental studies as well as of human observational studies, the effect of statins with bone metabolism seems to be individual instead of a general mechanism. Statins are categorized as lipophilic (atorvastatin, simvastatin, and lovastatin) and relatively hydrophilic (pravastatin and rosuvastatin) based on their intrinsic polar properties [28]. Due to differences in their inherent polarity and bone bioavailability, the individual bone effect might be varied. Considering the ability of both lipophilic and hydrophilic statins to inhibit the HMG-CoA reductase, it was proven that only the lipophilic statins prominently enhance BMP-2 expression to further promote osteoblasts differentiation [29,30]. Nowadays, lipophilic simvastatin seems to draw more attention by the majority of studies focused on bone effects derived from statin therapy. However, not only simvastatin, but also rosuvastatin and atorvastatin exhibit significant reduction in the new development of osteoporosis in our study. Regardless of statin potency, high-potency statins (atorvastatin and rosuvastatin) and moderate-potency statin (simvastatin) are more effective in ameliorating osteoporosis risk with HR 0.43, 0.68, and 0.85, respectively. A recent randomized, placebo-controlled study also demonstrated an improvement in total hip BMD after 48 weeks of rosuvastatin therapy among HIV-infected adults [31]. However, this is somewhat controversial to the findings of the JUPITER study, which showed no association between rosuvastatin and fracture risk. We speculated that rosuvastatin might exhibit some lipophilic manner although it inherits the hydrophilic property.

The pleiotropic osteoprotective effects of statins are proposed to be derived from several experimental studies. Mundy et al first showed that statins exerted beneficial effects on bone cells by augmenting osteoblast activity in vitro, mediated by enhanced expression of BMP-2, and subsequently increased bone formation [7]. Statins also inhibit the synthesis of mevalonate by HMG-CoA reductase to prevent the formation of isoprenoid precursors and further inhibit osteoclast activity via decrease in prenylation of GTP binding proteins.[32,33] In addition, statin down-regulates the expression of RANKL in the synoviocytes and affects the mevalonic acid pathway by up-regulating the expression of OPG, which inhibits the generation of osteoclasts [34]. It also inhibits osteoblastic apoptosis via the pathway of TGF-β/Smad3 [35,36]. However, due to a lack of direct studies in humans, bone-related biomarkers of statin therapy have been evaluated. A recent meta-analytical study indicated that statins increased the levels of osteocalcin, but have no significant effect on the bone-specific alkaline phosphatase and serum C-terminal peptide of type 1 collagen concentrations [27]. This implies the beneficial effect of statins on bone may be mainly attributed to bone formation rather than anti-resorption.

The strength of our study includes a longer follow-up period with a large sample size in real clinical practice, a specific ascertainment and larger number of the outcome events, a discovery of exposure-response relationship, and the influence of different statins. However, there were some limitations in this present study. First, this is a retrospective, observational study and the target outcome of osteoporosis was detected only using the coding system based on the Taiwan LHID dataset without any information of bone mineral density. Second, several confounders were not adequately adjusted such as lifestyle manners, physical activity, dietary intake of vitamin D and calcium supplements, concomitant use of other medicines, and genetic factors although the comorbidities and HRT in females were considered. Additionally, the association of an exposure-response relationship was due to accumulating doses without ascertainment of adherence.

## Conclusions

Our study suggests that long-term exposure to statins, especially high intensity ones, is associated with a reducing occurrence of new-onset osteoporosis in both genders. Thus, this finding was consistent with most previous studies, but controversy with the post-hoc analyses of RCTs. Therefore, conducting a prospective RCT to specifically elucidate the potential role of statins on bone is warranted.
